# Draft Genome Sequence of Arsenic-Resistant *Microbacterium* sp. Strain SZ1 Isolated from Arsenic-Bearing Gold Ores

**DOI:** 10.1128/genomeA.01183-17

**Published:** 2017-10-26

**Authors:** Zaratulnur Mohd Bahari, Zaharah Ibrahim, Jafariah Jaafar, Shafinaz Shahir

**Affiliations:** a Department of Biosciences and Health Sciences, Faculty of Biosciences and Medical Engineering, Universiti Teknologi Malaysia, Skudai, Johor, Malaysia; b Department of Chemistry, Faculty of Science, Universiti Teknologi Malaysia, Skudai, Johor, Malaysia

## Abstract

*Microbacterium* sp. strain SZ1 isolated from gold ores of a Malaysia gold mine was found to be highly resistant to arsenic. Here, we report the draft genome sequence of SZ1, which may provide further insights into understanding its arsenic resistance mechanism. In this draft genome, a complete set of *ars* operons and two additional scattered *ars* genes were encoded.

## GENOME ANNOUNCEMENT

Arsenic is a metalloid of global concern that primarily exists in two inorganic forms of severe toxicity, As(III) and As(V). Known arsenic-transforming bacteria have evolved various mechanisms of coping with arsenic toxicity either through an energy generation or detoxification mechanism that confers arsenic resistance (*ars* operon). The reduction of As(V) to As(III) through regulation of the *ars* operon increases the toxicity, mobility, and bioavailability of arsenic. Knowledge of the microorganisms capable of reducing As(V) would be of paramount importance to gain insights into its As detoxification mechanism and to find ways to limit its toxic effect in the environment. In this paper, we report the draft genome sequence of *Microbacterium* sp. strain SZ1 isolated from arsenic-bearing gold ores that tolerate As(V) at a half-maximal inhibitory concentration (IC_50_) of 140 mM (1). This is the first reported high-level arsenic-resistant microbe found in Malaysian arsenic-bearing gold ores with the ability to transform arsenic.

The genome sequencing of strain SZ1 was performed using the Illumina Genome Analyzer IIx (2 × 100 bp paired-end reads). The paired-end reads were assembled *de novo* using CLC Genomics Workbench 4.8 (CLC bio, Denmark). Structural RNA predictions were determined by using tRNAscan-SE 1.21 for tRNAs (2) and RNAmmer 1.2 for RNA (3). Prodigal version 2.60 was used to predict open reading frames (ORFs) in the draft genome of *Microbacterium* sp. strain SZ1 (4). The assembled genome sequence was annotated using Rapid Annotations using Subsystems Technology (RAST) (5) and Blast2GO (6). A total of 1,030,297,978 reads were generated at approximately 206-fold genome coverage and assembled into 48 contigs, with an *N*_50_ contig size of 267,243 bp. The genome assembly was 3,458,719 bp in length with 69.2% G+C content. A total of 145 tRNAs, 3 rRNAs, and 3,120 ORFs were identified.

Nine genes that are crucial for arsenic resistance are present in the SZ1 genome, which include an *ars* operon containing four arsenate reductases (*arsC*), an arsenical resistance protein (*ACR3*), a thioredoxin reductase (*arsT*), and an arsenical resistance operon repressor (*arsR*). The two additional genes, *arsC* and arsenic efflux pump (*arsB*), were located at the different contigs as scattered genes inside the genome (7). Neither the presence of a respiratory arsenate reductase (*arr*) nor an arsenite methyltransferase (*arsM*) gene was detected, suggesting the incapability of strain SZ1 to respire or methylate arsenic. In addition to arsenic resistance genes, strain SZ1 was also found to possess genes responsible for copper homeostasis, cobalt-zinc-cadmium resistance, mercuric reductase, siderophore production, and the uptake of selenite and selenate, thereby enhancing its versatility in tolerating toxic conditions.

### Accession number(s).

This whole-genome shotgun project has been deposited at DDBJ/ENA/GenBank under the accession number LPVV00000000. The version described in this paper is version LPVV01000000.
